# Transition to Virtual Learning During the Coronavirus Disease–2019 Crisis in Iran: Opportunity Or Challenge?

**DOI:** 10.1017/dmp.2020.142

**Published:** 2020-05-07

**Authors:** Soleiman Ahmady, Sara Shahbazi, Mohammad Heidari

**Affiliations:** Department of Medical Education, School of Medical Education, Shahid Beheshti University of Medical Sciences, Tehran, Iran; School of Management and Medical Education Sciences, Shahid Beheshti University of Medical Sciences, Tehran, Iran; Community-Oriented Nursing Midwifery Research Center, Shahrekord University of Medical Sciences, Shahrekord, Iran

**Keywords:** Covid-19, learning, education, virtual learning

## Abstract

COVID-19 is a respiratory disease that can spread from one person to person. This virus is a novel coronavirus that was first identified during an investigation into an outbreak in Wuhan, China. Iran’s novel coronavirus cases reached 17,361 on 17 March, while death toll reached approximately 1,135. Its first death was officially announced on 20 February 2020 in Qom. The 2019 coronavirus pandemic has affected educational systems around the world, Also in Iran, and led to the closure of face to face courses in schools and universities. Therefore, virtual education can be seen as a turning point in education of these days in Iran.

The 2019 coronavirus disease (COVID-19) is a respiratory disease that can spread from 1 person to another. The novel coronavirus, known as *severe acute respiratory syndrome (SARS-CoV-2)*, was first identified during an investigation of an outbreak in Wuhan, China.^1^ The disease has been spreading worldwide since its inception, as the World Health Organization declared an epidemic on March 11, 2020.^2^ COVID-19 cases in Iran reached 17 361 on March 17, while the death toll reached approximately 1135. The first death to COVID-19 was officially announced on February 20, 2020, in Qom.^3^

The COVID-19 pandemic has affected educational systems in countries around the world, including Iran, and led to the closure of face-to-face courses in schools and universities.^4^ Therefore, virtual education can be seen as a turning point in education in Iran due to COVID-19. Virtual learning offers many benefits that traditional college degree programs do not provide, such as accessibility from anywhere at any time, asynchronous discussions with classmates, immediate feedback on tests, and flexibility. However, despite the benefits of virtual learning, it is not always easy to implement.^5^

In Iran, virtual education for grade-school students is performed through scheduled programs through TV and mobile education through social media; and, in higher education, virtual instruction is performed through mobile messenger systems and learning management systems, such as NAVID, VESTA, and MOODLE.

In elementary and secondary school education in Iran, conditions of achieving virtual education during the crisis of COVID-19 are complex due to (1) cultural and social contexts, (2) lack of teachers’ preparation for virtual teaching, (3) lack of access to all infrastructures and equipment, (4) willingness to hold presence classes (ie, in-person), (5) impossibility of using mobile-based training for all age groups, (6) lack of access to smartphones, (7) insufficient literacy and technological capabilities, (8) inability to virtualize all courses, and (9) large number of learners and the limited time to prepare online courses.

The universities in Iran have adequate infrastructure, experience, and acceptances consistent with other global movements toward the context of virtual learning. This is an opportunity for higher education institutions to shift to virtual education. In spite of this, the main concern with the closing of higher education institutions is the impossibility of supporting courses that are fully operational and require in-person training and practice. Therefore, it seems that culture building and in-service training for professors are essential, and there is a need for the coordination and empathy of the relevant authorities and managers to plan accurately and coherently.
